# LncRNA DANCR promotes macrophage lipid accumulation through modulation of membrane cholesterol transporters

**DOI:** 10.18632/aging.205992

**Published:** 2024-07-02

**Authors:** Guo-Jun Zhao, Yu Wang, Jun-Hong An, Wan-Ying Tang, Xiao-Dan Xu, Kun Ren

**Affiliations:** 1 Affiliated Qingyuan Hospital, Guangzhou Medical University (Qingyuan People’s Hospital), Qingyuan 511518, Guangdong, China; 2 College of Medicine, Dali University, Dali 671003, Yunnan, China; 3 Department of Physiology, Institute of Neuroscience Research, Hengyang Key Laboratory of Neurodegeneration and Cognitive Impairment, Hunan Province Cooperative Innovation Center for Molecular Target New Drug Study, Hengyang Medical School, University of South China, Hengyang 421001, Hunan, China; 4 Department of Pathology, The First Affiliated Hospital of Anhui Medical University, Hefei 230032, Anhui, P.R. China; 5 College of Nursing, Anhui University of Chinese Medicine, Hefei 230012, Anhui, P.R. China; 6 Institute of Clinical Medicine, The Second Affiliated Hospital of Hainan Medical University, Haikou 570100, Hainan, P.R. China

**Keywords:** DANCR, miR-33a, lipid accumulation, cholesterol transport, macrophage

## Abstract

The progression of atherosclerosis (AS), the pathological foundation of coronary artery disease (CAD), is featured by massive lipid deposition in the vessel wall. LncRNAs are implicated in lipid disorder and AS, whereas the specific role of lncRNA DANCR in atherogenesis remains unknown. Here, we demonstrated that DANCR promotes macrophage lipid accumulation by regulating the expression of membrane cholesterol transport proteins. qPCR showed that compared to control groups, CAD patients and atherosclerotic mice had higher DANCR levels. Treating human THP-1 macrophages and mouse RAW264.7 macrophages with ox-LDL significantly upregulated the expression levels of DANCR. Oil Red O staining showed that the silence of DANCR robustly reduced, while overexpression of DANCR significantly increased the numbers and size of lipid droplets in ox-LDL-treated THP-1 macrophages. In contrast, the opposite phenomena were observed in DANCR overexpressing cells. The expression of ABCA1, ABCG1, SR-BI, and NBD-cholesterol efflux was increased obviously by DANCR inhibition and decreased by DANCR overexpression, respectively. Furthermore, transfection with DANCR siRNA induced a robust decrease in the levels of CD36, SR-A, and Dil-ox-LDL uptake, while DANCR overexpression amplified the expression of CD36, SR-A and the uptake of Dil-ox-LDL in lipid-laden macrophages. Lastly, we found that the effects of DANCR on macrophage lipid accumulation and the expression of membrane cholesterol transport proteins were not likely related to miR-33a. The present study unraveled the adverse role of DANCR in foam cell formation and its relationship with cholesterol transport proteins. However, the competing endogenous RNA network underlying these phenomena warrants further exploration.

## INTRODUCTION

Coronary artery disease (CAD), such as heart failure and stroke, is among the leading causes of morbidity and deaths around the globe [1]. Atherosclerosis (AS) is the pathological foundation of CAD. The deposition of macrophage foam cells within vessel walls is the hallmark of atherogenesis [2]. High-density lipoprotein cholesterol (HDL) can elicit lipid-lowering effects by mediating reverse cholesterol transport (RCT) [3]. RCT is a multi-step process that removes redundant cholesterol in macrophages to the liver for excretion [4]. Cholesterol efflux from macrophages mediated by ATP-binding cassette transporter A1/G1 (ABCA1/G1) and scavenger receptor class B type I (SR-BI) is the first and critical step of RCT [5]. Membrane ABCA1 mediates free cholesterol (FC) and phospholipid to extracellular lipid-free apoA-1 to form nascent HDL particles [6]. Subsequently, ABCG1 and SR-BI mobilize intracellular cholesterol to nascent HDL particles, creating mature HDL particles [7]. An ABCA1 gene mutation can cause a rare lipid metabolism disorder called Tangier disease, featuring severe deficiency of HDL-C in the plasma and massive accumulation of cholesteryl esters (CE) in diverse tissues of the body [8]. Wang et al. reported that in bone marrow-derived macrophages (BMMs) from ABCA1 knockout (ABCA1^-/-^) mice, the efflux of [^3^H]-cholesterol was inhibited entirely, compared with that from wild-type (WT) mice. Besides, injection of ABCA1^-/-^ BMMs into mice caused a significant reduction of [^3^H]-tracer levels in plasma and feces [9]. In ABCG1-overexpressing macrophages, the efflux of intracellular cholesterol to HDL was increased by nearly 40%. Furthermore, mice injected with ABCG1-overexpressing macrophages exhibited higher plasma [^3^H]-tracer levels and increased fecal [^3^H]-tracer excretion [9]. Compared to apoE^-/-^ mice, mice receiving ABCA1^-/-^apoE^-/-^ bone marrow displayed more severe lipid accumulation within atherosclerotic plaques [10]. Moreover, mice receiving ABCA1^-/-^ABCG1^-/-^ bone marrow had larger atherosclerotic lesions than those single knockout recipients [11]. Likewise, macrophage SR-BI can promote macrophage-to-feces RCT, lower plasma total cholesterol (TC) and triglycerides (TG), and alleviate atherosclerotic plaque formation [12]. In addition to ABCA1/G1 and SR-BI, lipid uptake mediated by CD36 and SR-A also contributes to macrophage foam cell formation. Targeted deletion of SR-A and CD36 can effectively prevent the formation of advanced necrotic lesions and promote plaque stability in hyperlipidemic mice [13]. Repression of CD36 and SR-A in atherosclerotic mice significantly alleviated macrophage foam cell formation and decreased the size of atherosclerotic plaques in apoE^-/-^ mice [14]. Thus, upregulation of ABCA1/G1 and SR-BI or downregulation of SR-A and CD36 is a practical approach to preventing foam cell formation and AS progression.

Macrophage miR-33a was demonstrated to impair *in vivo* RCT and aggravate atherosclerotic plaque formation by inhibiting ABCA1/G1 [15, 16]. In peritoneal macrophages from hypercholesterolemic mice, miR-33a levels were negatively associated with cellular cholesterol ester (CE) content and ABCA1 expression. Overexpression of miR-33a in macrophages significantly diminished the mobilization of intracellular cholesterol to extracellular apoA-1 or HDL [17]. Low-density lipoprotein receptor knockout (LDLR^-/-^) mice treated with anti-miR-33a oligonucleotides (anti-miR-33a) displayed increased HDL-cholesterol (HDL-C) levels in the plasma and improved macrophage-to-feces RCT *in vivo*. Additionally, miR-33a can inhibit lipophagy, i.e., autophagy-mediated degradation of intracellular lipid droplets (LDs), and therefore retard cholesterol efflux from macrophages [18]. LDLR^-/-^ mice administered with anti-miR-33a displayed enhanced autophagy in lipid-laden macrophages and reduced atherosclerotic plaque formation [19]. Emerging evidence has identified long non-coding RNAs (lncRNAs) as critical regulators of foam cell formation and AS development [20–23]. LncRNA DANCR was reported to regulate tumor progression by modulating cell proliferation [24], apoptosis [25], and inflammation [26]. However, the role of DANCR in macrophage lipid accumulation and atherogenesis is unknown. Here, the effects of DANCR on macrophage foam cell formation and its relationship with miR-33a were investigated, which may help deepen the understanding of the connections between lncRNAs and AS.

## MATERIALS AND METHODS

### Participants selection

Twelve patients (40 to 65 years of age) in the Department of Cardiology of the Qingyuan City People’s Hospital submitted to coronary angiography were enrolled. All participants did not have recent unstable angina, cancer, or liver and renal problems. They were divided into two groups according to the results of coronary angiography: the control group (n=6) and the atherosclerotic group (n=6). The procedures performed adhere to the tenets of the 1964 Helsinki Declaration [27].

### Mice treatment

12 male apoE^-/-^ mice (8 weeks old) and 6 wild-type C57BL/6 mice were bought from Zhaoqing CasGene Biotech. Co., Ltd (Guangdong, China). Mice were housed with free access to water and food under a 12 h light/dark cycle (24±2° C, 60% humidity). 6 apoE^-/-^ mice were fed a Western diet (0.3% cholesterol and 21% fat), and the rest mice were fed a chow diet. 12 weeks later, they were euthanized, and the blood and aorta and blood were collected to detect DANCR expression.

### PBMC isolation

PBMC was isolated from the blood of humans and mice, as previously described [28]. DPBS supplemented with sodium citrate buffer (1:1) was used to dilute EDTA-collected blood. Then, it was transferred into test tubes with 15 mL of Ficoll Paque Plus. After centrifugation at 1,000 rpm for 10 min, the PBMC fraction was collected using sterile pipettes, followed by another round of centrifugation at 600 rpm for 10 min. The supernatant was discarded, and cells were washed three times using DPBS without magnesium and calcium, supplemented with sodium citrate buffer and FBS, and centrifuged for 7 min at 250 g at RT. Afterward, the cells were resuspended in RPMI 1640 medium supplemented with FBS. A Nexcelcom Cell Counter was used to determine the total PBMC for each donor. Finally, PBMCs were cryopreserved for later use.

### Cell transfection

THP-1 monocytes and RAW264.7 macrophages were obtained from ATCC (Virginia, US). Cells were cultured in RPMI-1640 medium containing 10% FBS and 1% penicillin-streptomycin (37° C, 5% CO_2_). For experimental use, THP-1 monocytes were differentiated into macrophages by incubation with 160 ng/mL PMA (Sigma, USA) for 48 h and further treated with 50 μg/mL ox-LDL (Solarbio, Beijing, China) for 24 h to differentiate into foam cells. Before transfection, THP-1 macrophages were planted into 6-well plates containing 3~4×10^6^ cells/well.

For DANCR overexpression experiments, GeneChem (Shanghai, China) constructed a lentiviral overexpression vector (LV-DANCR). Cells were treated with 1×10^10^ TU/mL LV-DANCR using lipofectamine 2000 (Invitrogen, USA) for 48 h. A lentiviral vector that expressed GFP alone was used as a negative control (LV-NC). For DANCR knockdown experiments, the siRNA against DANCR (si-DANCR) was purchased from RiboBio (Guangzhou, China). Cells were incubated with 20 μM si-DANCR using lipofectamine 2000. The negative control group was transfected with a scrambled siRNA (si-NC). After 48 h, DANCR levels were measured by qPCR. For miR-33a mimic/inhibitor transfection, cells were transfected with 50 nM of miR-33a mimic/inhibitor or mimic/inhibitor control. At 48 h after transfection, cells were cultured in the RPMI-1640 medium containing 2% FBS. After another 48 h, miR-33a levels were detected by qPCR. Supplementary Table 1 listed the relevant sequence used in this study.

### Oil red O staining

Oil Red O staining was used to analyze the lipid accumulation in THP-1 macrophages. After transfection, cells were washed thrice in PBS and fixed at 4% paraformaldehyde for 10 min. Following dehydration with 60% isopropanol for 2 min at 37° C, a 0.3% Oil Red O solution was added to stain cellular lipid droplets for another 10 min. The pictures were observed and captured under optical microscopy. Image-Pro Plus software was used to quantify the degree of lipid accumulation. The Oil Red O-positive area was recorded and expressed as fold change, as previously described [29].

### Cholesterol uptake assay

Dil-ox-LDL uptake assay was used to measure the cholesterol uptake capacity by THP-1 macrophages. Cells were co-incubated with Dil-ox-LDL (10 μg/μL) at 37° C for 4 h in a dark environment. Then, DAPI was added to counterstain the cells and track the nucleus. Photographs were taken using an optical microscope [30].

### Cholesterol efflux assay

NBD-cholesterol efflux assay was conducted according to the previous literature [31]. THP-1 macrophages were cultured with 5 μM fluorescent NBD-cholesterol (Invitrogen, USA) at 37° C for 6 h. Afterward, the cells were thricely washed in PBS and equilibrated in RPMI-1640 medium for 2 h. Then, the medium was supplemented with apoA-I (25 μg/mL) or HDL (50 μg/mL) at 37° C for 4 h.

0.3 M NaOH solution was used to lyse the cells for 15 min. The fluorescence-labeled cholesterol (green color) was visualized using an inverted fluorescence microscope, and fluorescence intensity was determined using Image-Pro Plus software. The cholesterol efflux was calculated following the equation: [fluorescence in medium/fluorescence in (cell+medium)] ×100%.

### Quantitative real-time PCR (qPCR)

After cells were transfected, TRIzol reagent (Invitrogen, USA) was used to extract the total RNA per the manufacturer’s instructions. Then, RNase-free DNase I was added to remove genomic DNA. Nanodrop 3000 (Thermo Fisher Scientific, USA) assessed the extracted RNA’s purity. All RNA samples’ absorption ratios (OD_260_/OD_280_) ranged from 1.8 to 2.0. Afterward, the TaqMan MicroRNA Reverse Transcription Kit (Applied Biosystems, USA) was utilized to reverse transcribe 2 μg total RNA for cRNA synthesis. Using SYBR Green PCR Master Mix (Applied Biosystems), a StepOnePlus™ Real-Time PCR System (Applied Biosystems, USA) was utilized to conduct qPCR reactions. Supplementary Table 1 lists the gene-specific primers, and Shanghai General Biotech Co., Ltd synthesized all the primers. U6 was used as an internal control for miR-33a and DANCR, and the other genes used GAPDH as a control. The relative changes in gene expression were determined using the 2^-ΔΔCt^ method.

### Western blot

After cells were transfected, the total proteins were obtained using a mixture of RIPA buffer (Beyotime, Beijing, China) and PMSF at a ratio of 94:6. A BCA assay kit (CWBIO, Peking, China) was used to detect the protein concentrations. An equivalent amount of protein (approximately 20 μg/lane) was added onto the 8% or 10% SDS-PAGE for electrophoresis. Following fractionation at 120 V for 90 min in gel running buffer, the proteins were transferred to 0.45 μm PVDF membranes (Merck Millipore, Darmstadt, Germany). Then, they were blocked with 5% fat-free dry milk dissolved in TBST at 4° C for 4 h and incubated with primary antibodies (1:1,000, Affinity Biosciences, Jiangsu, China) at 4° C with gentle shaking overnight. After rinsing with TBST thricely (10 min each), membranes were immuno-blotted with corresponding secondary antibody (1:5,000, Abcam) for 2 h at 4° C. Finally, the bands were visualized, and the relative protein levels were assessed using the Quantity One software.

### Statistical analysis

All data were represented as mean ±SD from at least three independent experiments. They were compared using Student’s unpaired t-test or a one-way ANOVA, followed by Tukeys’ post hoc test via GraphPad Prism 10.0.3 software. *P* < 0.05 was considered as statistical significance.

### Data availability statement

The data are available from the corresponding author upon reasonable request.

## RESULTS

### DANCR expression is significantly higher in CAD patients and atherosclerotic mice

**Figure 1:**
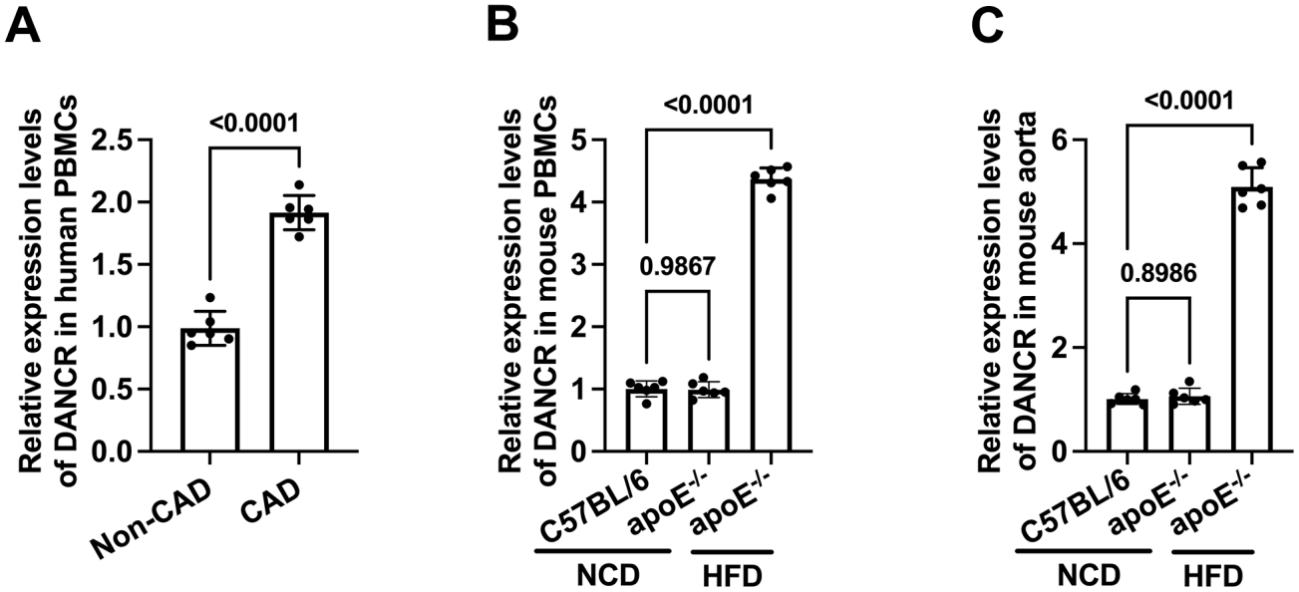
**The expression of DANCR in CAD patients and HFD-fed apoE^-/-^ mice.** (**A**) Detection of DACNR expression in PBMCs isolated from non-CAD and CAD patients. (**B**) Detection of DACNR levels in PBMCs from NCD- or HFD-fed or HFD-fed apoE^-/-^ mice. (**C**) Detection of DACNR expression in the aorta from NCD- or HFD-fed apoE^-/-^ mice. NCD, normal chow diet; HFD, high-fat diet. n=6.

We first detected the expression of DANCR in CAD patients and HFD-fed apoE^-/-^mice using qPCR. The results showed that compared to non-CAD patients, the DANCR levels in PBMCs of CAD patients were significantly higher (Figure 1A). Similarly, HFD-fed apoE^-/-^mice displayed increased expression of DANCR in PBMCs and aortas compared to chow diet-fed mice (Figure 1B, 1C), indicating that DANCR may aggravate atherogenesis.

### Ox-LDL upregulates DANCR expression in both human and murine macrophages

**Figure 2:**
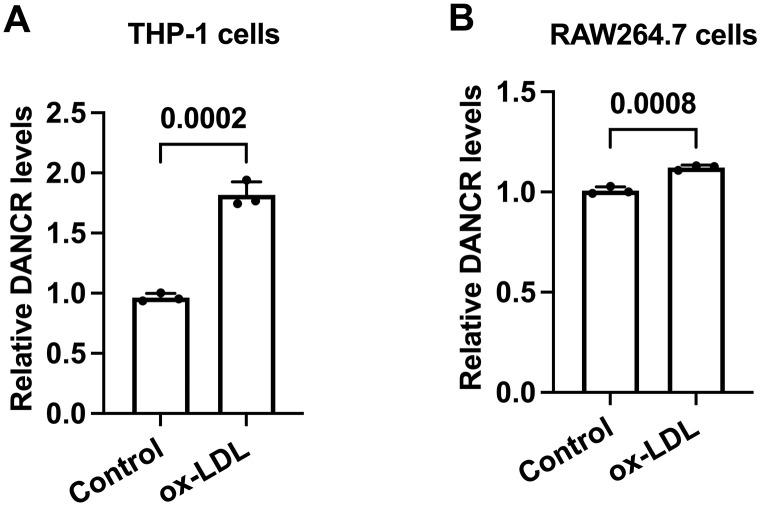
**The expression of DANCR is increased in ox-LDL-treated human THP-1 macrophages and mouse RAW264.7 macrophages.** (**A**, **B**) THP-1 cells were incubated with 160 ng/mL PMA for 48 h to differentiate into macrophages. Then, THP-1-derived macrophages or RAW264.7 macrophages were treated with 50 μg/mL ox-LDL for 24 h. qPCR was used to detect the expression of DANCR. All results are presented as the mean ± SD from three independent experiments performed in triplicate. n=3.

Ox-LDL is a well-known factor to drive foam cell formation *in vitro* [32]. To preliminary unravel the link between DANCR and lipid accumulation, we assessed the DANCR levels in ox-LDL-treated human and murine macrophages. Cells were treated with PBS or ox-LDL for 48 h, then qPCR was used to detect the DANCR levels. The results showed that ox-LDL upregulated DANCR levels in human and murine macrophages (Figure 2A, 2B), indicating that DANCR may adversely affect macrophage lipid accumulation.

### DANCR promotes lipid accumulation in THP-1 monocytes-derived macrophages

**Figure 3:**
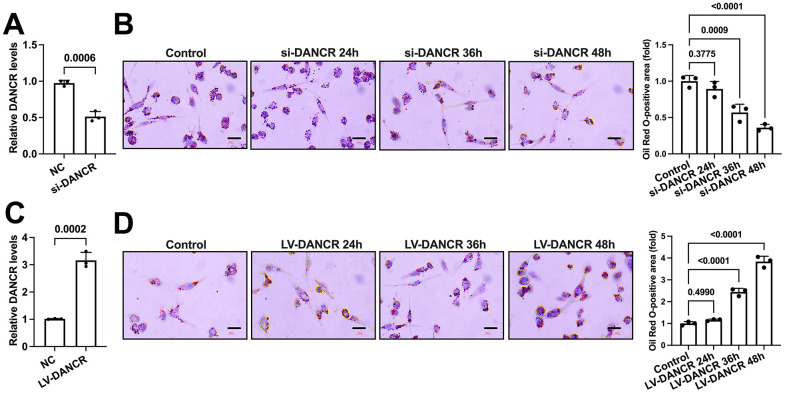
**DANCR promotes lipid accumulation in THP-1 macrophages.** (**A**) THP-1 macrophages were transfected with 20 μM si-DANCR or the negative control for 48 h. Transfection efficiency was determined by qPCR. (**B**) THP-1 macrophages were treated with 50 μg/mL ox-LDL for 24 h. Then, they were transfected with 20 μM si-DANCR for 24 h, 36 h, and 48 h, respectively. Oil Red O staining was used to detect lipid droplets. Image-Pro Plus software calculated Oil Red O-positive areas. (**C**) THP-1 macrophages were transfected with 1×10^10^ TU/mL LV-DANCR or the negative control for 48 h. Transfection efficiency was determined by qPCR. (**D**) THP-1 macrophages were treated with 50 μg/mL ox-LDL for 24 h. Then, they were transfected with 1×10^10^ TU/mL LV-DANCR for 24 h, 36 h, and 48 h, respectively. Oil Red O staining was used to detect lipid droplets. Image-Pro Plus software calculated Oil Red O-positive areas. All results are presented as the mean ± SD from three independent experiments performed in triplicate. Scale bar=200 μm.

Next, we detected whether DANCR could influence lipid accumulation in THP-1 macrophages. Cells were incubated with ox-LDL and transfected with si-DANCR or LV-DANCR for 48 h. It is observed that si-DANCR treatment significantly reduced DANCR expression by approximately 45%, while LV-DANCR transduction increased DANCR levels by nearly 2.2-fold (Figure 3A, 3C). Subsequently, si-DANCR or LV-DANCR were transfected into macrophages for different durations (0 h, 24 h, 36 h, 48 h). Oil Red O staining showed that the lipid deposition in ox-LDL-treated THP-1 macrophages was robustly attenuated after si-DANCR treatment time-dependently (Figure 3B). Conversely, transfection with LV-DANCR at various times gradually increased the numbers of lipid droplets (Figure 3D). Thus, DANCR contributes to lipid accumulation in THP-1 macrophages.

### DANCR promotes lipid uptake and suppresses the efflux of cholesterol in THP-1 macrophages

**Figure 4:**
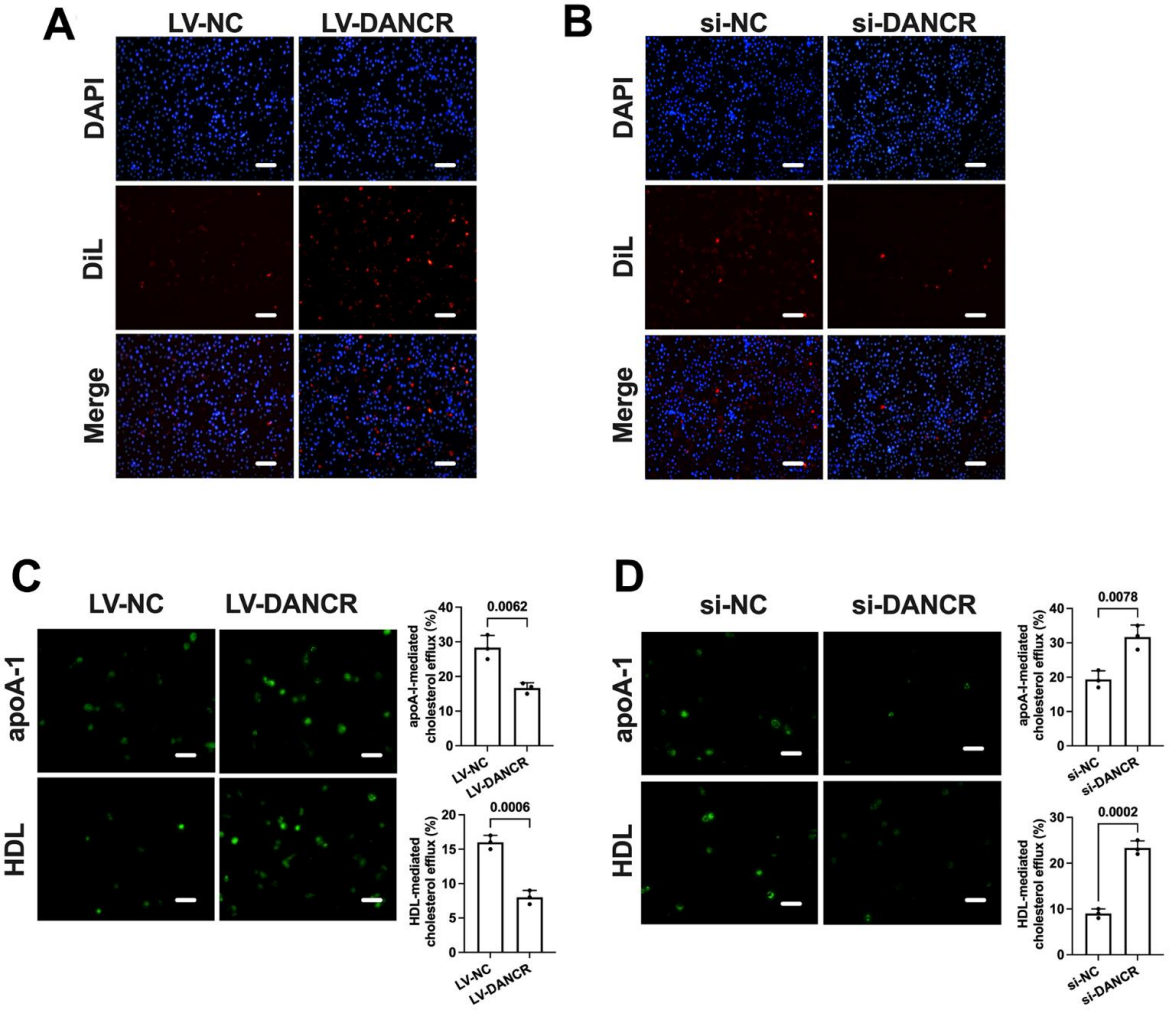
**DANCR promotes lipid uptake and inhibits cholesterol efflux in THP-1 macrophages.** THP-1 macrophages were treated with 50 μg/mL ox-LDL for 24 h. Then, they were transfected with 1×10^10^ TU/mL LV-DANCR or 20 μM si-DANCR or their corresponding control for 48 h. (**A**, **B**) Cells were incubated with 10 μg/μL Dil-ox-LDL at 37° C for 4 h. The representative fluorescent images of Dil-ox-LDL uptake were photographed (200×). (**C**, **D**) Cells were loaded with 5 μM NBD-cholesterol for 6 h. Then, the medium was supplemented with 25 μg/mL apoA-I or 50 μg/mL HDL for 4 h. The representative fluorescent images of the NBD cholesterol burden in THP-1 macrophage-derived foam cells were photographed (200×). All results are presented as the mean ± SD from three independent experiments performed in triplicate. Scale bar=50 μm.

The conversion of macrophages to lipid-laden foam cells is related to the balance of cholesterol uptake and efflux. Next, we detected the effects of DANCR suppression and overexpression on DiL-ox-LDL uptake and NBD-cholesterol efflux in human THP-1 macrophages. Ox-LDL-treated THP-1 macrophages were transduced with si-DANCR or LV-DANCR for 48 h. As shown in Figure 4, uptake of Dil-ox-LDL by THP-1 macrophages was robustly increased by overexpression of DANCR and decreased by the silence of DANCR, respectively (Figure 4A, 4B). At the same time, apoA-1/HDL-mediated cholesterol efflux was significantly suppressed by LV-DANCR transfection and augmented by si-DANCR transfection, respectively (Figure 4C, 4D). These findings indicate that increased lipid uptake and decreased cholesterol efflux are responsible for the positive effects of DANCR on lipid accumulation in THP-1 macrophages.

### DANCR upregulates SR-A and CD36 levels and downregulates ABCA1/G1 and SR-BI levels in THP-1 macrophages

**Figure 5:**
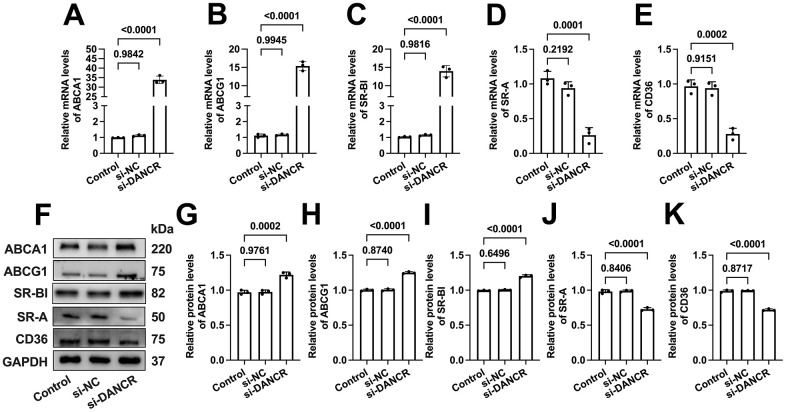
**Effects of DANCR inhibition on the expression of membrane cholesterol transporters in ox-LDL-treated THP-1 macrophages.** THP-1 macrophages were treated with 50 μg/mL ox-LDL for 24 h. Then, they were transfected with 1×10^10^ TU/mL LV-DANCR or 20 μM si-DANCR or their corresponding control for 48 h. (**A**–**E**) Detection of the mRNA levels of ABCA1, ABCG1, SR-BI, SR-A, and CD36 by qPCR. (**F**–**K**) Detection of the protein levels of ABCA1, ABCG1, SR-BI, SR-A, and CD36 by Western blot. All results are presented as the mean ± SD from three independent experiments performed in triplicate. n=3.

**Figure 6:**
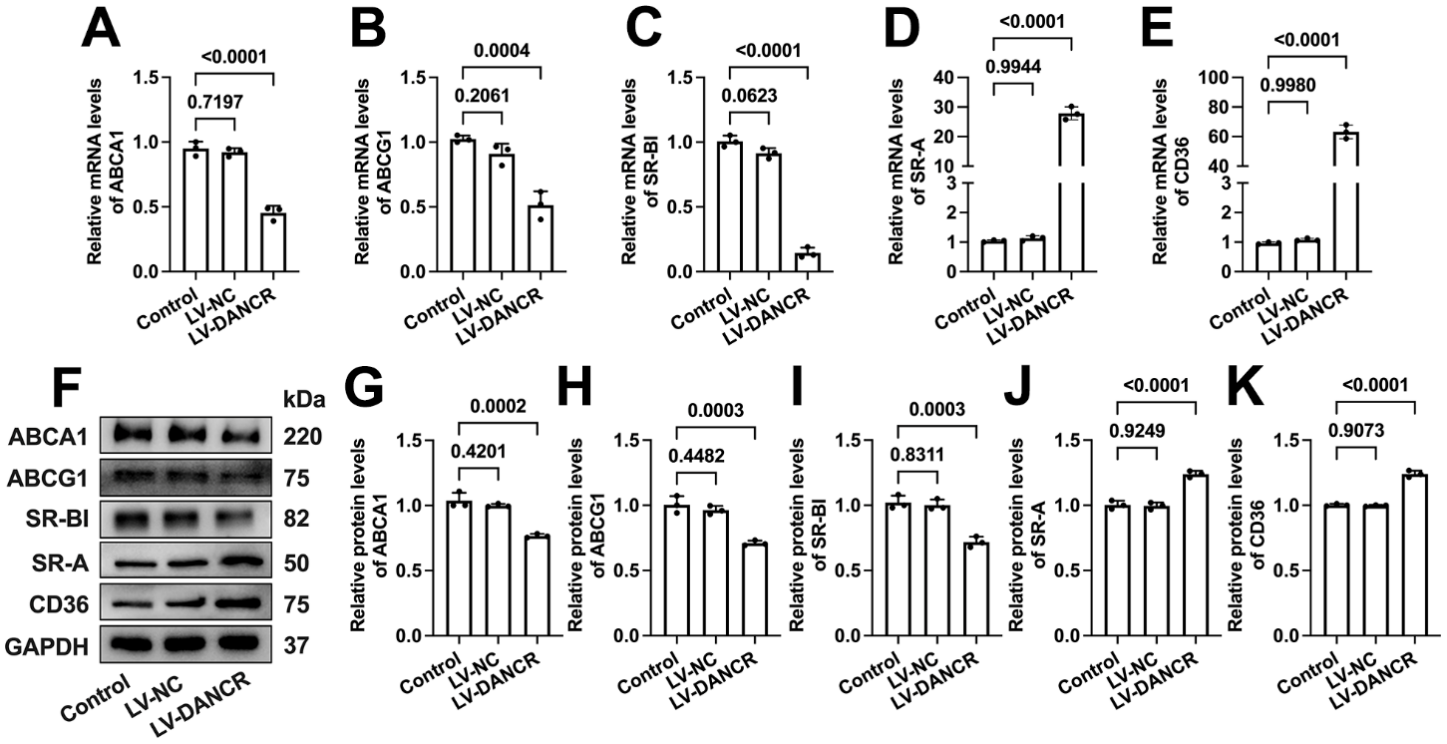
**Effects of DANCR overexpression on the expression of membrane cholesterol transporters in ox-LDL-treated THP-1 macrophages.** THP-1 monocytes were incubated with 160 ng/mL PMA for 48 h to differentiate into macrophages. Then, they were treated with 50 μg/mL ox-LDL for 24 h, followed by transfection with 1×10^10^ TU/mL LV-NC or LV-DANCR 48 h. (**A**–**E**) Detection of the mRNA levels of ABCA1, ABCG1, SR-BI, SR-A, and CD36 by qPCR. (**F**–**K**) Detection of the protein levels of ABCA1, ABCG1, SR-BI, SR-A, and CD36 by Western blot. All results are presented as the mean ± SD from three independent experiments performed in triplicate. n=3.

ABCA1 can mediate the initial transport of free cholesterol (FC) to apoA-1, promoting the formation of nascent HDL particles [33]. Subsequently, these HDL particles accept continued FC efflux facilitated by ABCG1 and SR-BI for further maturation [34]. Besides, CD36 and SR-A can mediate ox-LDL uptake by macrophages, significantly contributing to macrophage lipid accumulation [35, 36]. To explore the potential mechanisms beneath the effects of DANCR on lipid uptake and efflux, we next assessed the impact of DANCR silence or overexpression on the expression of these five membrane cholesterol transporters. As expected, si-DANCR transfection significantly increased the ABCA1/G1 and SR-BI expression. Meanwhile, SR-A and CD36 levels were markedly decreased (Figure 5). Moreover, the mRNA and protein levels of ABCA1/G1 and SR-BI were reduced after DANCR overexpression in lipid-laden THP-1 macrophages. In contrast, CD36 and SR-A expression was sharply increased upon LV-DANCR transfection (Figure 6). These results indicate that regulation of these membrane cholesterol transporters underlies DANCR-induced lipid accumulation in THP-1 macrophages.

### DANCR modulates membrane cholesterol transporter levels in a miR-33a independent manner

**Figure 7:**
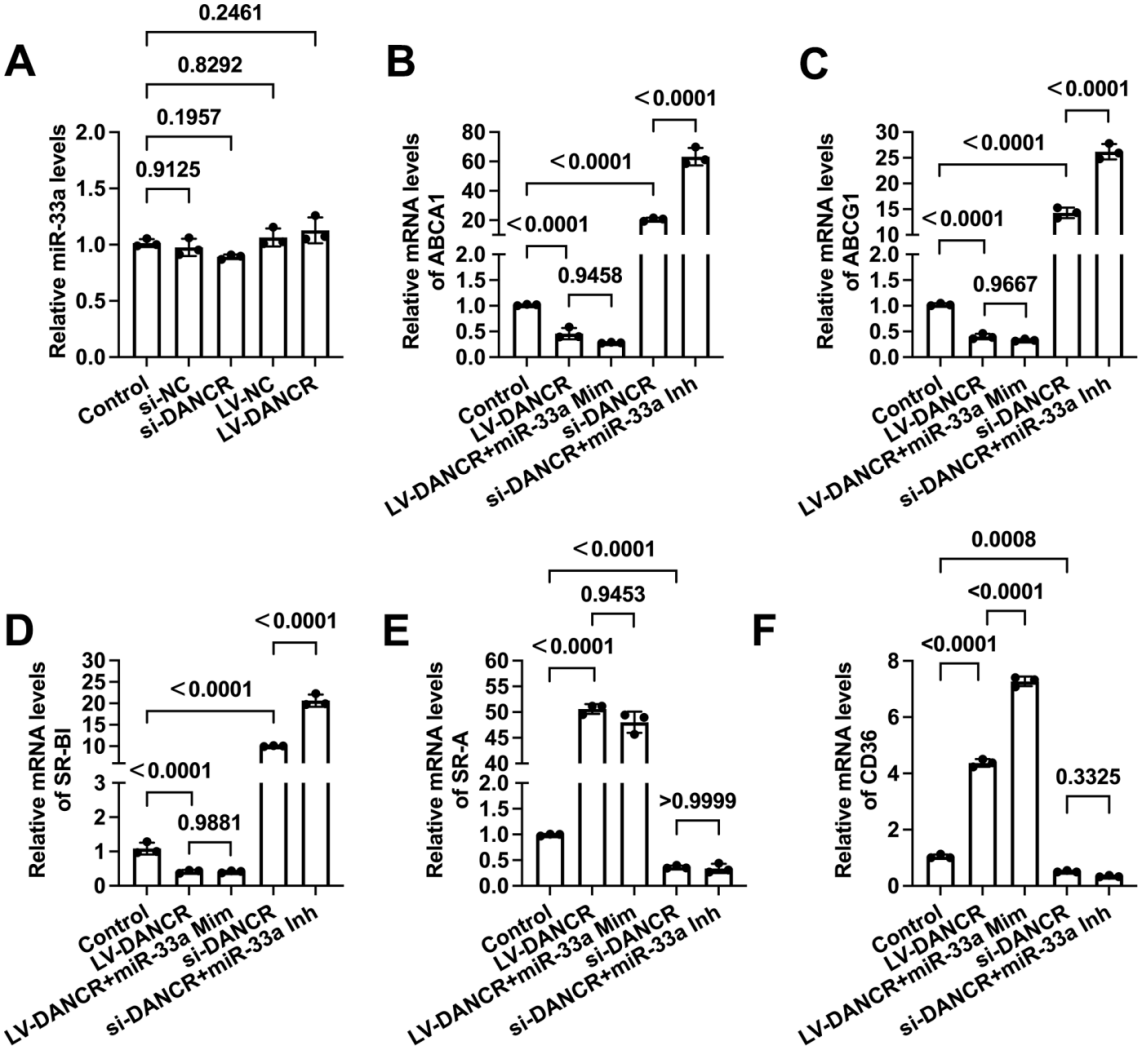
**DANCR regulates the expression of membrane cholesterol transporters in a miR-33a-independent manner.** THP-1 macrophages were treated with 50 μg/mL ox-LDL for 24 h. (**A**) Cells were transfected with 20 μM si-DANCR, 1×10^10^ TU/mL LV-DANCR, or their corresponding control for 48 h. miR-33a levels were determined by qPCR. (**B**–**F**) Cells were transfected with 20 μM si-DANCR, 1×10^10^ TU/mL LV-DANCR, and 50 nM miR-33a mimic (Mim) /inhibitor (Inh) for 48h. Then, qPCR was conducted to determine the expression of ABCA1, ABCG1, SR-BI, SR-A, and CD36. All results are presented as the mean ± SD from three independent experiments performed in triplicate.

miR-33a can promote macrophage lipid accumulation by targeting ABCA1 and ABCG1 [37, 38]. Previous research has unraveled that DANCR can exert various biological effects by serving as a molecular sponge for miR-33a [39, 40]. Next, we determined whether DANCR regulates the levels of membrane cholesterol transporters through miR-33a in THP-1 macrophages. As shown in Figure 7A, the expression of miR-33a was not influenced by either si-DANCR or LV-DANCR transfection. Next, cells were treated with si-DANCR or LV-DANCR, followed by miR-33a mimic/inhibitor transduction. The results showed that miR-33a mimic treatment amplified the upregulation of CD36 expression induced by LV-DANCR (Figure 7F), while the expression of the rest of the cholesterol transporters was unchanged (Figure 7B–7E). Furthermore, the positive effects of si-DANCR on macrophage ABCA1, ABCG1, and SR-BI were more evident after miR-33a inhibitor transfection (Figure 7B–7D). At the same time, si-DANCR-induced down-regulation of SR-A and CD36 was not influenced after miR-33a inhibitor transfection (Figure 7E, 7F). These findings indicated that DANCR does not likely regulate the expression of membrane cholesterol transporters by sponging miR-33a in THP-1 macrophages.

## DISCUSSION

Although tremendous progress has been made in treating AS, various side effects are still present in the long-term treatment plans [2]. The formation of atherosclerotic lesions is featured by the massive deposition of lipid-loaded macrophages within the vessel wall. Targeting macrophage lipid accumulation effectively treats hyperlipidemia and AS [41, 42]. Here, we found that ox-LDL, a risk factor in developing AS, can upregulate DANCR levels in human and murine macrophages. DANCR overexpression promoted macrophage lipid accumulation, whereas suppression of DANCR caused the opposite effects, suggesting the adverse role of this lncRNA in foam cell formation. DANCR may be a potent stimulator of hyperlipidemia and atherogenesis.

LncRNAs are involved in macrophage lipid accumulation by regulating ABCA1/G1-mediated cholesterol efflux. LncRNA MeXis can promote ABCA1 gene transcription via interaction with DDX17 and subsequent activation of LXRα. In THP-1 macrophages, MeXis overexpression increased ABCA1 expression and enhanced apoA-1-mediated cholesterol efflux [43]. Sun et al. found that inhibition of exosomal LOC100129516 derived from mesenchymal stem cells activated the PPARγ/LXRα/ABCA1 pathway and prevented detrimental lipid deposition in ox-LDL-treated THP-1 macrophages [44]. LncRNA DAPK-IT1 can downregulate ABCA1/G1 levels and diminish apoA-1/HDL-mediated cholesterol efflux by sponging miR-590-3p, leading to less formation of THP-1 macrophage-derived foam cells [45]. LncRNA THRIL can interact with Forkhead box protein O1(FOXO1) and decrease the transcription of ABCA1/G1, causing damage to cholesterol efflux and more lipid deposition in macrophages [46]. However, whether lncRNAs can regulate macrophage lipid accumulation by affecting SR-BI has not been reported. Our results showed that the expression of ABCA1/G1, SR-BI, and the capacity of cholesterol efflux was significantly elevated by DANCR suppression and decreased by DANCR overexpression, respectively, suggesting that down-regulation of cholesterol efflux is responsible for macrophage lipid accumulation induced by DANCR.

In addition to cholesterol efflux, excessive cholesterol uptake is another promoter in macrophage lipid accumulation. LncRNAs are critical regulators of CD36-mediated lipid uptake in macrophages and hepatocytes. Ning et al. found that ox-LDL can increase lncRNA MALAT1 expression, which recruits β-catenin to binding sites on the CD36 promoter, leading to increased lipid uptake in THP-1 macrophages [47]. LncRNA UCA1 can increase oxidative stress and CD36 expression by sponging miR-206, and inhibition of UCA1 significantly reverses ox-LDL-induced macrophage foam cell formation [48]. Another research showed that MALAT1 could promote free fatty acids (FFA)-induced hepatocyte lipid accumulation. Specifically, MALAT1 upregulates the expression of aryl hydrocarbon receptor nuclear translocator (ARNT) through binding with miR-206, increasing CD36 expression and lipid uptake [49]. Moreover, Kim et al. reported that PPARα could augment the expression of lncRNA 3930402G23Rik (G23Rik) by binding to its promoter region, further decreasing CD36 levels and attenuating hepatic lipid accumulation [50]. So far, the association between lncRNAs and SR-A-mediated cholesterol uptake has not been well disclosed. We found that DANCR up-regulation significantly increased CD36 and SR-A expression and subsequent uptake of Dil-ox-LDL. In contrast, suppression of DANCR exerted the opposite effects, indicating that enhanced cholesterol uptake is involved in DANCR-induced macrophage lipid accumulation.

miR-33a can influence atherogenesis and cholesterol metabolism by targeting ABCA1/G1 in macrophages [17]. The expression of miR-33a is conversely related to ABCA1/G1 in monocytes of patients with primary hypertension [51]. Antisense inhibition of miR-33a in hepatocytes and macrophages significantly upregulated ABCA1 expression and accelerated cholesterol efflux. Injection of Western diet-fed mice with anti-miR-33a oligonucleotides resulted in elevated HDL-C levels in the plasma [52]. Urolithin A (UA), the most abundant ellagitannin, can mitigate lipid deposition in mouse RAW264.7 macrophages via miR-33a inhibition and enhancement of ABCA1/G1-mediated cholesterol efflux [53]. A furostanol bisglycoside Methyl protodioscin can decrease miR-33a levels by inhibiting SREBP1c and SREBP2 transcription, leading to upregulation of ABCA1-mediated cholesterol efflux [54]. The lncRNA CHROME-miR-33 axis can enhance ABCA1 expression, cholesterol efflux, and the formation of nascent HDL particles, alleviating lipid deposition in macrophages [55]. Recently, DANCR was found to exert various biological activities by inhibiting miR-33a. Yang et al. reported that DANCR decreased miR-33a levels, inhibited apoptosis of glioma cells, and increased epithelial-mesenchymal transition [56]. In osteosarcoma cell lines, DANCR up-regulated the expression of kinase AXL by acting as a sponge of miR-33a, leading to cell proliferation, migration, and metastasis [40]. Feng et al. determined that miR-33a can inhibit cell proliferation and insulin production of INS-1 cells, effects that can be rescued by DANCR overexpression [57]. Unexpectedly, our results showed that DANCR overexpression or inhibition did not change miR-33a levels in THP-1 macrophages. Transfection of miR-33a mimic did not influence the effects of LV-DANCR on ABCA1/G1, SR-A, and SR-BI expression. Furthermore, the negative effects of si-DANCR on SR-A and CD36 levels were not changed by miR-33a inhibitor treatment. These observations indicate that DANCR regulates the expression of membrane cholesterol transporters in a miR-33a-independent manner. According to previous studies, NF-κB is a key regulator of the five membrane cholesterol transporters. Li et al. reported that the Qing-Xue-Xiao-Zhi formula increased ABCA1/G1-mediated cholesterol efflux by suppressing the TLR4/NF-κB pathway in murine macrophages [58]. Hu et al. found that silencing ZAP70 inhibited the NF-κB signaling pathway and increased ABCA1/G1- and SR-BI-mediated cholesterol efflux from T cells [59]. Besides, Hyun et al. observed that metformin could attenuate inflammation and decrease CD36 and SR-A levels via down-regulation of NF-κB translocation in RAW264.7 macrophages [60]. Interestingly, DANCR was found to activate the NF-κB signaling and cause cisplatin resistance in glioma cells [61]. These pieces of evidence imply that DANCR may regulate the expression of membrane cholesterol transporters by activating NF-κB. More investigations are warranted to verify this hypothesis.

In conclusion, this study demonstrated that DANCR can decrease ABCA1/G1- and SR-BI-mediated cholesterol efflux and increase SR-A- and CD36-mediated cholesterol influx, contributing to macrophage foam cell formation. Moreover, these effects are miR-33a independent. DANCR may become a novel, promising target for AS therapy.

## Supplementary Material

Supplementary Table 1
