# Supplementary material for: LncRNA DANCR promotes macrophage lipid accumulation through modulation of membrane cholesterol transporters
Source: Aging (Albany NY). 2024 Jul 2;16(18):12510–24. doi: 10.18632/aging.205992 (PMC11466482; doi:10.18632/aging.205992)
Supplement: Supplementary Table 1 [file aging-16-205992-s001.pdf]

SUPPLEMENTARY TABLE

Supplementary Table 1. The primer sequences used in this study.

Gene	Sequences
DANCR (Human)	Forward, 5'-ACTCACTCACTCACTCACT-3';<br>Reverse, 5'-GCCTCTGTATACTATTCTTGCCA-3'
DANCR (Mouse)	Forward, 5'-CGCGAGACACAAAGTCCTCT -3';<br>Reverse, 5'-CTGGAACCTCCGTCTTCTCG -3'
miR-33a (Human)	Forward, 5'-CCTCATAAGCGGTGCATTGTA-3';<br>Reverse, 5'-TATGCTTGTTCTCGTCTCTGTGTC-3'
ABCA1 (Human)	Forward, 5'-GCTCAGTGGGATGGATGGCAAAG-3';<br>Reverse, 5'-CTCCGTCTGGCAATTAGCAGTCTC-3'
ABCG1 (Human)	Forward, 5'-TCTCGGTGGATGAGGTGGTGTC-3';<br>Reverse, 5'-GCTGGGCTTCCGTGAGGTTATTATC-3'
SR-BI (Human)	Forward, 5'-AGCAAGGTTGACTTCTGGCATTCC-3';<br>Reverse, 5'-TGTAGAACTCCAGCGAGGACTCAG-3'
SR-A (Human)	Forward, 5'-GACACTGATAGCTGCTCCGAATCTG-3';<br>Reverse, 5'-AAACACGAGGAGGTAAAGGGCAATC-3'
CD-36 (Human)	Forward, 5'-GGAAGTGATGATGAACAGCAGCAAC-3';<br>Reverse, 5'-TGTCCTCAGCGTCCTGGGTTAC-3’
si-DANCR (Human)	Forward, 5'-CUGCAUUCCUGAACCGUUATT-3';<br>Reverse, 5'-UAACGGUUCAGGAAUGCAGTT-3’
miR-33a mimics	Forward, 5'-GUGCAUUGUAGUUGCAUUGCA-3';<br>Reverse, 5'-CAAUGCAACUACAAUGCACUU-3’
miR-33a inhibitor	Forward, 5'-UGCAAUGCAACUACAAUGCAC-3';
GAPDH (Human)	Forward, 5'-GGAGCGAGATCCCTCCAAAAT-3';<br>Reverse, 5'-GGCTGTTGTCATACTTCTCATGG-3'
U6 (Human)	Forward, 5'-GCTTCGGCAGCACATATACTAAAAT-3';<br>Reverse, 5'-CGCTTCACGAATTTGCGTGTCAT-3'
U6 (Mouse)	Forward, 5'-CTCGCTTCGGCAGCACATATACT-3';<br>Reverse, 5'-ACGCTTCACGAATTTGCGTGTC-3'
